# Increased levels of choline metabolites are an early marker of docetaxel treatment response in BRCA1-mutated mouse mammary tumors: an assessment by *ex vivo* proton magnetic resonance spectroscopy

**DOI:** 10.1186/s12967-015-0458-4

**Published:** 2015-04-09

**Authors:** Jack JA van Asten, Riyas Vettukattil, Tessa Buckle, Sven Rottenberg, Fijs van Leeuwen, Tone F Bathen, Arend Heerschap

**Affiliations:** Department of Radiology and Nuclear Medicine, Radboud University Medical Center, Nijmegen, The Netherlands; Department of Circulation and Medical Imaging, Faculty of Medicine, Norwegian University of Science and Technology (NTNU), Trondheim, Norway; Interventional Molecular Imaging Laboratory, Department of Radiology, Leiden University Medical Center, Leiden, The Netherlands; Department of Molecular Pathology, The Netherlands Cancer Institute- Antoni van Leeuwenhoek hospital (NKI-AvL), Amsterdam, The Netherlands

## Abstract

**Background:**

Docetaxel is one of the most frequently used drugs to treat breast cancer. However, resistance or incomplete response to docetaxel is a major challenge. The aim of this study was to utilize MR metabolomics to identify potential biomarkers of docetaxel resistance in a mouse model for BRCA1-mutated breast cancer.

**Methodology:**

High resolution magic angle spinning (HRMAS) ^1^H MR spectroscopy was performed on tissue samples obtained from docetaxel-sensitive or -resistant BRCA1-mutated mammary tumors in mice. Measurements were performed on samples obtained before treatment and at 1-2, 3-5 and 6-7 days after a 25 mg/kg dose of docetaxel. The MR spectra were analyzed by multivariate analysis, followed by analysis of the signals of individual compounds by peak fitting and integration with normalization to the integral of the creatine signal and of all signals between 2.9 and 3.6 ppm.

**Results:**

The HRMAS spectra revealed significant metabolic differences between sensitive and resistant tissue samples. In particular choline metabolites were higher in resistant tumors by more than 50% with respect to creatine and by more than 30% with respect to all signals between 2.9 and 3.6 ppm. Shortly after treatment (1-2 days) the normalized choline metabolite levels were significantly increased by more than 30% in the sensitive group coinciding with the time of highest apoptotic activity induced by docetaxel. Thereafter, choline metabolites in these tumors returned towards pre-treatment levels. No change in choline compounds was observed in the resistant tumors over the whole time of investigation.

**Conclusions:**

Relative tissue concentrations of choline compounds are higher in docetaxel resistant than in sensitive BRCA1-mutated mouse mammary tumors, but in the first days after docetaxel treatment only in the sensitive tumors an increase of these compounds is observed. Thus both pre- and post-treatment tissue levels of choline compounds have potential to predict response to docetaxel treatment.

## Background

Breast cancer remains the most common type of malignancy and is also the second most common cause of cancer deaths in females [1]. The main treatment modalities include surgery, radiotherapy and systemic therapy. Among the chemotherapeutic agents, docetaxel has well established benefits in the treatment of breast cancer [2,3]. Unfortunately, resistance to chemotherapy remains a common problem during treatment. Many patients do not respond to docetaxel or will cease to respond after the initiation of therapy, either because of an inherent or an acquired resistance, resulting in the development of progressive disease [4]. Molecular and metabolic biomarkers may identify docetaxel resistant and sensitive subjects and stratify patients for better treatment options. However, thus far there are no reliable methods to predict the response to docetaxel prior to treatment or to identify the patients who will most likely benefit from therapy. A shortcoming of previous attempts to identify markers for response may be that tumors were not subjected to chemo therapeutic drugs when sampled for analysis, or treatment was given a few weeks before sampling (neoadjuvant trials). Relevant factors, like altered metabolism or apoptosis may be easier to monitor shortly after docetaxel-induced stress.

Alterations in the metabolic composition of cells and tissues represent the ultimate response of biological systems to genetic and environmental changes. Thus, high throughput analysis of small-molecular metabolites (metabolomics) may uncover changes in metabolite concentrations as indicators of cellular and tissue response to external stimuli [5-10]. This offers unique opportunities to elucidate drug response mechanisms and to identify response biomarkers. Magnetic resonance spectroscopy (MRS) has the potential to detect metabolic changes in a non-invasive way. For instance, in ^1^H MR spectra obtained from breast tumors *in vivo* the composite signal of the N-methyl protons of all choline compounds (total choline or tCho) is often increased compared to normal tissue. This may serve as a biomarker to detect cancer *in vivo* [11]. More specifically, this signal can be used as an indicator of response to neoadjuvant chemotherapy in locally advanced breast cancer [12]*.* The overlapping signals of the different choline compounds can be resolved in ^1^H High Resolution Magic Angle Spinning (HRMAS) MRS from intact tissue samples, measured at higher magnetic field strengths [13].

To study docetaxel resistance, we used a genetically engineered mouse model for BRCA1-mutated breast cancer [14]. In this model mice develop spontaneous tumors that highly resemble their human counterpart [15]. Moreover, the docetaxel responses seen in these animals mimic the response variability seen in patients [16]. The aim of this study was to identify a metabolic marker for the response to docetaxel treatment by using HRMAS MRS on tissue samples obtained from sensitive and resistant tumors that develop in the BRCA1 model. Such a marker could be useful to evaluate the effectiveness of docetaxel treatment.

## Materials and methods

### Animals

Sections of T*23 sensitive (N = 14) and T*23 resistant (N = 20) tumors from syngeneic mice (K14cre;Brca1^F/F^;p53^F/F^), were orthotopically transplanted into a mammary fat pad of female WT mice as described by Beekman et al. [15]. At week three to four after transplantation, therapeutic intervention and sampling of tissue for HRMAS MRS was started (tumor size of 7–10 mm in diameter). The treatment and monitoring period of tumor bearing mice were as described [15]. In brief, after day 0, the animals received 25 mg/kg docetaxel (Taxotere, Aventis Pharma BV, diluted to 2.5 mg/ml in 0.9% saline) by intravenous tail vein injection. Tumor response was monitored at day 0 (no treatment) and 1-2 days, 3-5 days and 6-7 days post treatment. Biopsies for HRMAS MRS experiments were harvested and snapshot frozen in liquid nitrogen immediately after sacrificing the mice (n ≈ 4-7 for each monitoring point). The treatment efficacy was evaluated by volumetric tumor measurements (caliper measurements and computed tomography) and by immunohistochemical staining for apoptosis (TUNEL) [15]. All animal experiments were performed in accordance with Dutch animal welfare regulations and were approved by the local ethics committee DEC (Dier Experimenten Commissie) from The Netherlands Cancer Institute- Antoni van Leeuwenhoek hospital (NKI-AvL), Amsterdam, The Netherlands.

### NMR experiments

Multiple tumor sections from the different groups (n = 23 resistant, n = 19 sensitive; in 8 cases two tissue specimens were obtained from the same xenograft) were analysed by HRMAS MRS using a Bruker DRX 500 MHz spectrometer, equipped with a ^1^H/^13^C HRMAS probe head, optimized for ^1^H sensitivity. The biopsies were gently thawed and cut (sample weight: 4.5 to 11 mg) to fit in a 12ul sphere (2.8 mm diameter) of a zirconia rotor. D_2_O was added to fill up the sphere and to lock the B_0_ field. The sphere of the MAS rotor (4 mm diameter) was closed by a teflon insert and sealed with a Kel-F cap and then placed at an angle of 54°44” with respect to the main magnetic field. Rotating the sample under this angle averages out the line broadening effects of chemical shift anisotropy and dipolar coupling, typical for spectra of samples in semi-solid state. NMR spectra were acquired at 4°C at a magic angle spinning rate (masr) of 4 kHz, employing the 1D ^1^H NMR CPMG (Carr Purcell Meiboom Gill) [17] sequence (T2 filter/TR = 30/5000 ms) [18]. The 90° pulse was optimized per sample ranging from 6.2 to 9.5 μs. A T_2_ filter of 30 ms was used to suppress the contribution of macromolecular components with short transverse relaxation times (T_2_ times below 30 ms). An interval of 1 rotor period (1/masr) between the 180° pulses enabled optimal suppression of J-modulation effects and chemical shift anisotropy, and minimized the contribution of diffusion to relaxation processes. The water resonance was suppressed by presaturation. With an excitation bandwidth of 6000 Hz collected in 16 K complex points the acquisition time was 1.36 seconds, and with 256 transients collected every 5 seconds, the total measurement time was ~25 minutes. The whole procedure from the start of thawing until the end of acquisition was less than 45 minutes.

### Data processing and analysis

The MR spectra, apodized with a 0.3 Hz exponential filter, were analyzed in two different ways. First, the correlation between metabolic profiles and treatment effect was assessed by multivariate data analysis, performed in MATLAB (Version 7.9.0; The Math Works, Natick, MA, USA). To exclude variations in lipid signals arising from the mammary fat pad, the spectral region between 2.9–4.5 ppm containing the majority of the small molecular metabolites was selected in the multivariate analysis. In addition the region from 3.6 to 3.7 ppm was excluded to discard contaminating ethanol signals from the analysis. Spectra were normalized by setting the total signal integral of this spectral area to a constant value (=1) for all spectra to minimize differences in sample weight. Unsupervised principal component analysis (PCA) was performed using PLS_Toolbox v5.8.3 (Eigenvector Research, Manson, WA, USA). PCA reduces the dimensionality of the data and summarizes the structure of multiple MR spectra in score plots and loading profiles. The variance structure of the data is explained by linear combinations of the variables called principal components (PCs). The first PCs will be in the direction explaining most of the variance in the data set. In the score plot of the PCs, samples with a similar metabolic profile will cluster. Loading profiles display the importance of each variable within the PC.

In the second approach, the spectra were subjected to a peak fitting procedure. Based on the results of the PCA analysis we focused on the spectral range from 2.9 – 3.6 ppm. The metabolite signals of glycerophosphocholine (GPC), phosphocholine (PCho), choline (Cho), and creatine (Cr) were fitted with Lorentzian lines, using Bruker Topspin software. The spectral areas determined by peak fitting were normalized to the signal integral of Cr. Since Cr, as an internal reference, could be influenced by the docetaxel treatment [19], the spectral areas of the choline signals were also normalized to the total area (integral) of all signals in the 2.9 – 3.6 ppm region. The results were statistically analyzed by a two-tailed unpaired *t*-test.

## Results

### Metabolic differences between docetaxel resistant and sensitive tumors before treatment

**Figure 1:**
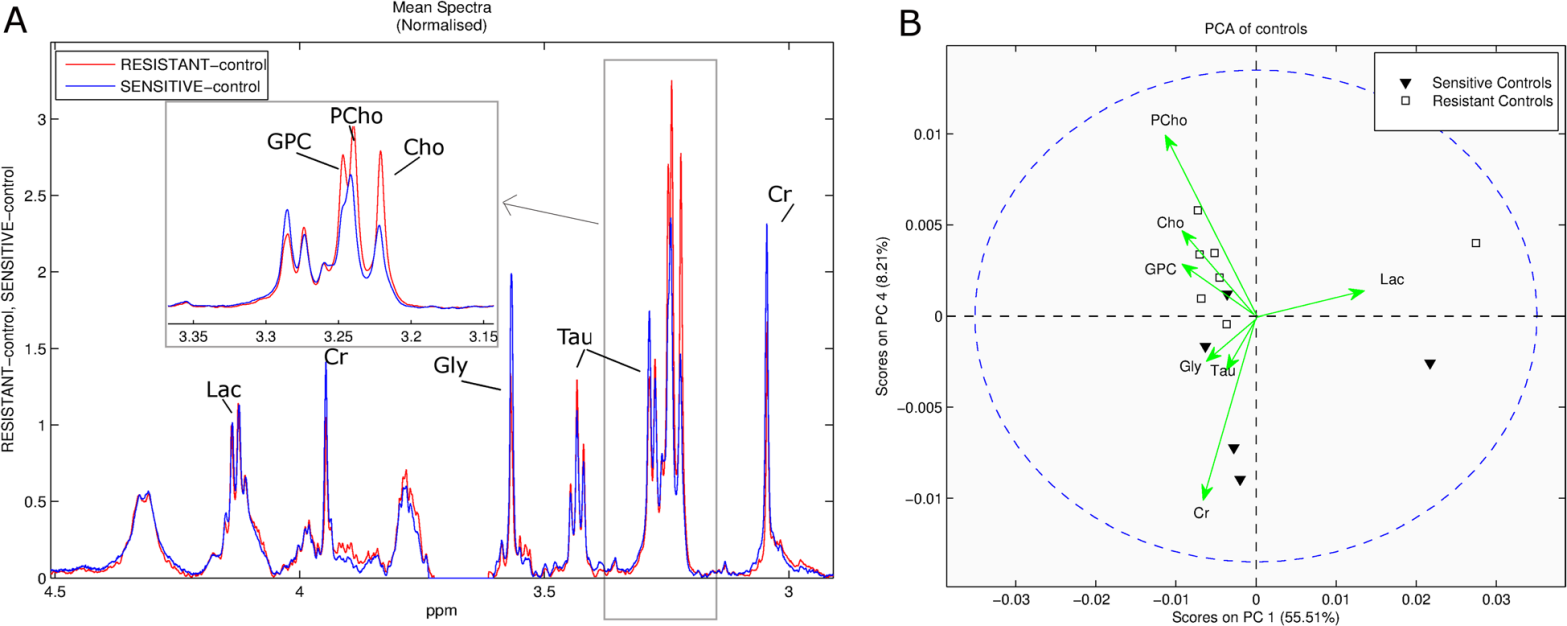
^**1**^ **H HRMAS spectra and Principal Component Analysis (PCA) of control tumor samples. A**: The mean spectra of resistant tumor (red) show increased choline compounds compared to those of sensitive tumors (blue). **B**: Biplot [42] showing PCA analysis of HRMAS spectra. Docetaxel sensitive samples are shown with black triangle and resistant samples with squares. Arrows are drawn based on the loading plots to show the important metabolites responsible for the demarcation between the groups. Resistant samples have higher levels of GPC, PCho and Choline.

A comparison of spectra obtained by ^1^H HRMAS MRS of resistant and sensitive breast cancer tissue shows some clear spectral differences between 3.1 and 3.4 ppm (Figure 1A). Analysis by PCA of spectra obtained before the start of treatment (day 0), revealed that the tumors indeed were metabolically distinct (Figure 1B). All choline groups including phosphocholines (PCho), free choline (Cho) and glycerophosphocholines (GPC) were higher in resistant than in sensitive tumors. In contrast, PCA indicated that sensitive control tumors had higher levels of glycine (Gly), taurine (Tau) and Cr. For other metabolites with signals in the spectral region between 2.9 and 4.5 ppm, such as myo-inositol and ethanolamine, no differences could be detected between the two tumor lines.

**Figure 2:**
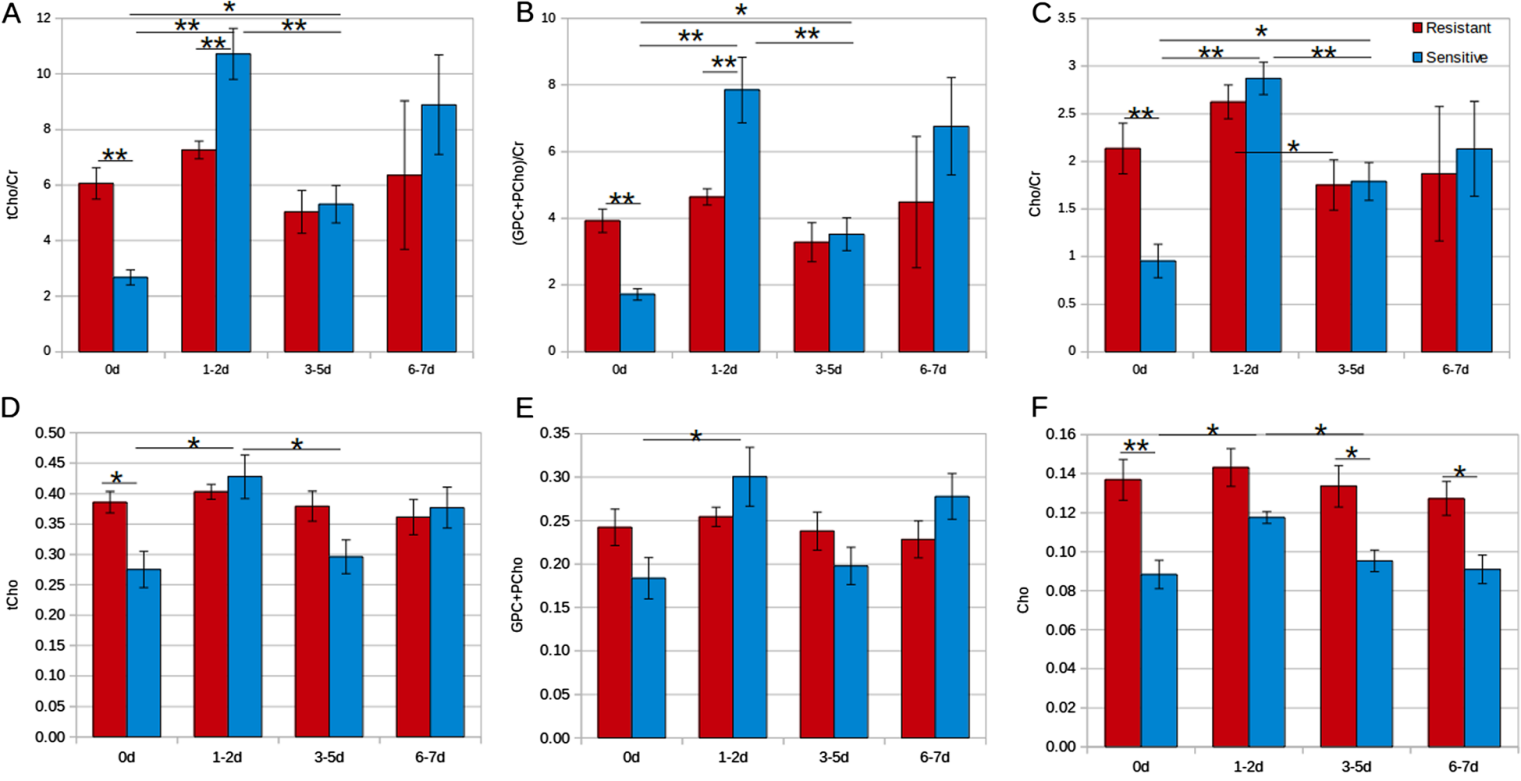
**Choline compound over creatine signal ratios (A-C) and normalized choline compound integrals (D-F) of docetaxel resistant and sensitive tumor tissue samples pre- and post-treatment.** Docetaxel resistant and sensitive tumor samples were monitored pretreatment at day 0 and after treatment at time points 1-2, 3-5 and 6-7 days. Statistical significant differences are indicated with an asterisk (*p < 0.05, **p < 0.01). The number of samples of resistant and sensitive tumor tissue for the different time points were: [7,5], [7,5], [5,5] and [4,4].

Spectral peak fitting confirmed that the sensitive tumors had significantly lower levels of choline metabolites than resistant tumors at day 0 (Figure 2A-F and Table 1A (p < 0.05)).

**Table 1:** **P-values of metabolite differences**

**A) P values of metabolite differences between resistant and sensitive tumors**											
**P (Resist-Sens)**	**tCho/Cr**			**(GPC+PC)/Cr**	**Cho/Cr**		**tCho**	**GPC+PC**		**Cho**	
**0d**	0.001			0.003	0.005		0.026	-		0.006	
**1-2d**	0.025			0.041	-		-	-		-	
**3-5d**	-			-	-		-	-		0.029	
**6-7d**	-			-	-		-	-		0.033	
**B) P values of metabolite differences between monitored time points after docetaxel treatment**											
**P (time)**	**0d**	**1-2**	**3-5d**		**0d**	**1-2d**	**3-5d**		**0d**	**1-2d**	**3-5d**
**tCho/Cr**	0.0009	-	0.003	**(GPC+PC)/Cr**	0.004	-	0.013	**Cho/Cr**	0.0001	-	0.006
**tCho/Cr**	-	0.021	-	**(GPC+PC)/Cr**	-	0.028	-	**Cho/Cr**	-	0.023	-
								**Cho/Cr**	-	*0.041*	-
**tCho**	0.020	-	0.033	**(GPC+PC)**	0.038	-	-	**Cho**	0.019	-	0.019

Significance of choline metabolite signal differences (p-values), corresponding with Figure 2.

**A**:between the resistant and sensitive tumor tissue samples of metabolite signals with respect to the creatine signal and with respect to the total integral of all signals between 2.9 and 3.6 ppm. If no number is presented the differences were not significant.

**B**:between the measurement time points, assessing treatment effect of the resistant and sensitive tumor tissue samples. Numbers for sensitive (regular) and resistant (italic) tumor tissue represent the p-values between the overlapping time points.

### Effect of treatment

**Figure 3:**
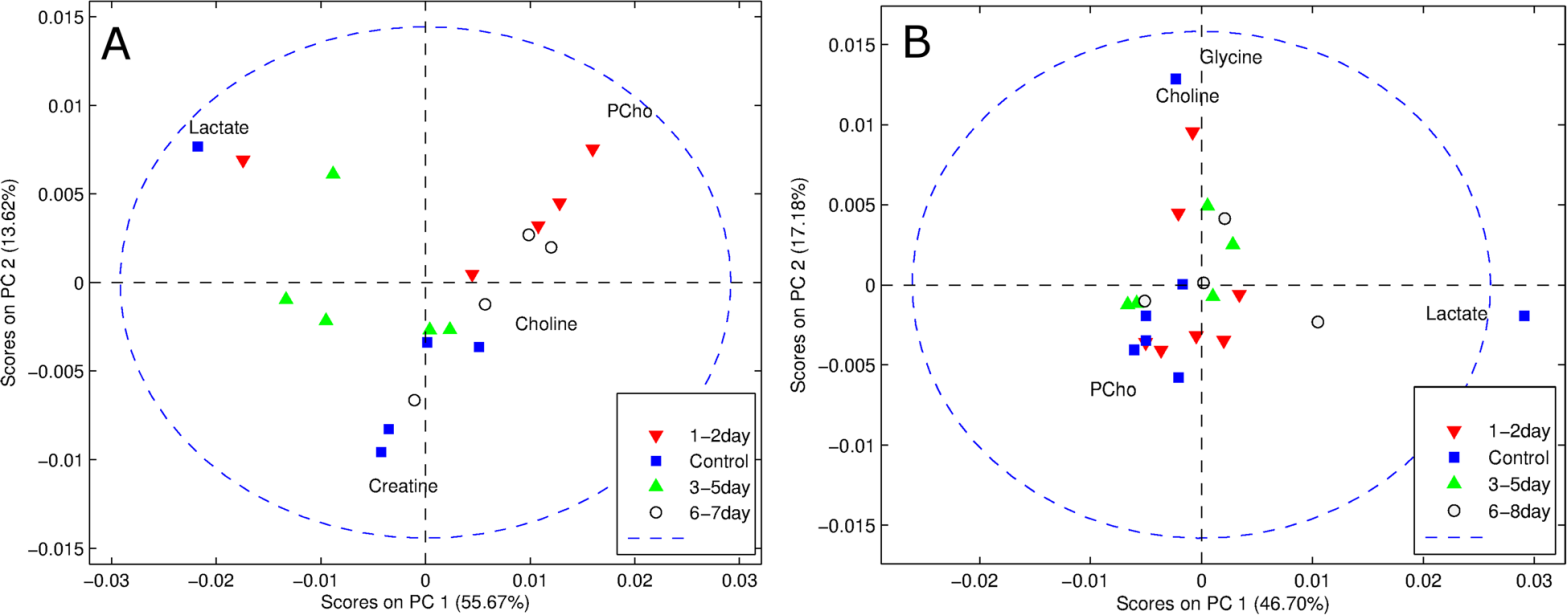
**Principal Component Analysis (PCA) biplots** **[** 42 **]** **of the sensitive (A) and resistant (B) strains of mouse models.** Plots show the differences in the metabolic profiles depending on the number of days after the administration of docetaxel. The sensitive strain shows distinct clusters based on the number of days after treatment **(A)** while the resistant strain does not **(B)** show such clusters.

As previously reported, tumor growth after docetaxel treatment significantly decreased in the sensitive tumors, and TUNEL staining showed a significant increase of apoptotic cells in these tumors with the highest increase at day 1-2, while this was not the case in the resistant tumors [15]. The biopsies from sensitive and resistant strains were evaluated separately by principal component analysis. PCA score plots of the sensitive strains (Figure 3A) showed a trend towards clustering based on the post treatment interval. The metabolic profiles of sensitive tumor samples obtained within 48 hours of treatment were characterized by higher levels of PCho compared to untreated controls. At 3-5 days post treatment, samples contained higher amounts of lactate, but for samples obtained at 6-7 days post treatment there was no difference with pre-treatment and 1-2 day post treatment groups. PCA of the resistant strain showed no tendency toward clustering based on the post treatment intervals (Figure 3B).

To assess the significance of changes in choline metabolites detected in the exploratory analysis by PCA, the signals of GPC, PCho, Cho were quantified with respect to Cr and with respect to the total signal integral of the spectral region from 2.9 to 3.6 ppm (Figure 2). The p-values of the significant differences are listed in Table 1B. For sensitive tumor tissues, the ratios of total choline (tCho), (GPC + PCho) and of Cho to Cr were significantly increased (p < 0.005) at one to two days post-treatment (Figure 2A-C). In agreement with this observation also a significant increase was found for the integrals of these choline metabolite signals normalized to the total signal integral (2.9 - 3.6 ppm) (Figures 2D-F). However, at three to five days post-treatment, the signal integral of these metabolites compared to that of creatine and to the total signal integral (over 2.9 – 3.6 ppm) decreased for the sensitive tumors (Figure 2A-F). The choline metabolite levels relative to creatine at three to five days post-treatment were still higher compared to the pre-treatment levels (Figure 2A-C), but for these levels relative to the total signal integral (2.9-3.6 ppm) the values were not significantly different from those before treatment (Figure 2D-F). At six to seven days post treatment there were no further significant changes in the relative levels of choline metabolites for the sensitive group compared to the levels at three to five days in this group.

In contrast to these responses for the sensitive tumors, the signal levels of all choline compounds in the docetaxel resistant tumors did not change significantly during the entire time course after treatment (Figure 2). The mice in which two specimens were obtained for HRMAS showed comparable results within the standard deviation of the subgroups, except for one sensitive specimen at day 7, that had lower choline signals than the mean. Glycine and taurine levels did not change during the post-treatment period in both subgroups except for an increase of about 13% of the normalized taurine level at days 3 – 7 in the docetaxel sensitive group (p = 0.034).

## Discussion

In this study we used a well-documented mouse breast cancer model that develops spontaneous tumors which share key features with BRCA1- associated breast cancer in humans [20]. We observed that tissue of docetaxel sensitive tumors in this model have a lower level of choline compounds compared to the resistant tumors. However, after docetaxel treatment the sensitive tumors showed a transient increase in these compounds, while their level remained stable in the resistant tumors. These differences in metabolite content and response to treatment were recorded by HRMAS MRS of tissue samples, which is a method that can be employed in the clinic to assess metabolites in fine-needle biopsies [18,21].

One of the major obstacles in the treatment of breast cancer is the effective application of chemotherapy. Selecting treatments based on the clinical and molecular characteristics of the tumor have the potential for an individualized, more effective, and less toxic therapy. However, in clinical decision making involving tumor treatment with docetaxel a good biomarker that can predict response to this drug is lacking [4]. Previously, we have tested if Annexin-V (^99m^Tc-AnxV) uptake values in our mouse tumor model, as measured by Single Photon Emission Computed Tomography (SPECT), could serve this purpose, but the uptake values for sensitive and resistant tumors were overlapping [15]. Here we identified an elevated level of choline metabolites (GPC, PCho, Cho) as a potential biomarker to differentiate between tumors sensitive and resistant to docetaxel treatment. Converting this result into a clinically useful response indicator still has a long way to go, as it requires the identification and validation of reliable threshold values for pretreatment choline containing compounds in patients, that may be able to distinguish between responders and non-responders. The application of HRMAS MRS in studies using fine-needle or core biopsy samples from breast cancer patients under docetaxel treatment, may be useful to establish such cutoff values [21]. Successful development and implementation of metabolomics based detection of docetaxel resistant breast cancers may allow clinicians to plan optimal therapies for the right patient.

After administration of docetaxel, there was a clear time dependent change in the metabolic patterns of sensitive tumors as compared to resistant tumors. Multivariate analysis revealed an increase in choline metabolites (PCho) in the sensitive tumors, which was more clearly observed by metabolite signal quantification. This showed increased Cho and (GPC + PCho) signals normalized to Cr or to the total signal integral between 2.9 and 3.6 ppm at 1 – 2 days post-treatment. In the following days this increase was (partly) reversed. This clearly identifies the increased levels of choline compounds as a early marker for the response to docetaxel treatment in the BRCA-1 tumor model.

Apoptosis or programmed cell death is observed at an enhanced rate in tumors responding to various cytotoxic drugs that have different mechanisms of action [22]. It is well-known that docetaxel also induces apoptosis in cancer cells although the precise molecular mechanism is still unclear [23-26]. Whereas choline deficiency is found to induce apoptosis in some cell systems [27,28], the results of our study on treatment sensitive BRCA-1 mutated tumors indicate that increased apoptotic activity by docetaxel treatment is associated with an increase in choline metabolites. There are two plausible explanations for this increase. First, the metabolic changes are merely reflecting an unspecific association with apoptosis or cell death. The second possibility is that apoptosis induced by docetaxel is mediated by alterations in metabolic pathways involving choline compounds. For instance it was demonstrated that the induction of apoptosis by different agents in two different cell lines (HL-60 and CHO-K1) increased the level of CDP-choline [29]. Changes in PCho and phospho-ethanolamine (PE) were not consistent across different apoptotic inducers. In a study of human neutrophils undergoing TNF-alpha induced apoptosis, an increase in PCho using MRS and HPLC was observed [30]. This effect on PCho content may indicate activation of phospholipases associated with apoptosis or a selective failure of phosphatidylcholine synthesis. An association between increased choline compounds and apoptosis shortly after therapy was also found in an ex vivo MR study of rat glioma under gene therapy [31]. Cancer therapy by histone deacetylase inhibition, known to induce apoptosis [32], increases PCho levels in tumor cells and xenografts [25,33]. Since the changes in choline metabolites are insignificant in resistant BRCA-1 tumors, there is a good reason to hypothesize that the observed changes in choline containing metabolites are linked to the docetaxel response of the sensitive tumors and possibly add to its mechanism of action including its well-known effect on microtubules [4]. The role of choline containing compounds in breast cancer development and progression is widely recognized. Their tissue levels are used as biomarkers of response to therapy [11]. Unlike most of the studies reporting a reduction in choline containing metabolites in response to therapy of breast cancer [34-37], in this study we observed an increase in choline metabolites, as an early response after chemotherapy in the docetaxel sensitive tumors. This discrepancy may be due to different times between metabolic MR observation and therapy or to the dosage of the used drug (vide infra) and to its specific interaction with (selected) breast tumor types [25,38]. More in line with our findings are the observation of increased GPC and PCho levels in MCF7 breast cancer cells after histone deacytelase inhibition, which was assigned to an elevated choline transporter and choline kinase activity [39].

In this study we used a single animal model with a specific genetic pattern with a single dosage regimen and hence can impart certain limitations. Although animal models offer a practical solution to explore the tumour biology, their ability to mimic the extremely complex aspects of human carcinogenesis, progression and pathophysiology are limited. Therefore it is necessary to validate these findings in further clinical studies; especially where other factors such as tumor heterogeneity and combination chemotherapies are involved. In cell culture studies it was shown that the concentration of docetaxel will influence its mechanism of action at the molecular level [40]. A low dosage caused aberrant mitosis followed by late necrosis where as a higher dosage resulted in mitotic arrest and apoptosis. Therefore, it is needed to further evaluate the response to different dosage regimens in animal model studies. The inherent limitation of ex-vivo studies include potential metabolic changes occurring during biopsy procedures, but all biopsy samples in this study were snapshot frozen to minimize such changes. Owing to overlap of GPC and PCho signals in some of the proton MR spectra, we combined the integrals of these signals. With ^31^P HRMAS MRS it is possible to resolve resonances for these individual metabolites, which could improve the specificity of treatment prediction [41].

## Conclusions

In conclusion, this study shows that ^1^H HRMAS MRS can distinguish between docetaxel sensitive and resistant BRCA1-mutated mouse mammary tumors because they are metabolically distinct. Furthermore, in docetaxel sensitive tumors an increase in choline containing metabolites is observed at 1-2 days after the initiation of therapy, which corresponds to the time of maximum apoptotic activity in these tumors [15]. Relative pre-treatment tissue concentrations of choline compounds are higher in docetaxel resistant than in sensitive tumors. These results indicate that metabolomics by HRMAS MRS may predict and monitor chemotherapy response in docetaxel treatment and might enable a more efficient breast cancer treatment procedure.
